# Assessment of the Potential of Bitter Melon (*Momordica charantia*) and Squash (*Cucurbita pepo*) as Rootstocks for Enhancing Drought Tolerance in Cucumber

**DOI:** 10.3390/plants15131996

**Published:** 2026-06-27

**Authors:** Aslı Kacar, Zekiye Erdogan, Gokhan Erdogan, Hayri Ustun, Aylin Kabas, Duoduo Wang, Selman Uluisik

**Affiliations:** 1 Faculty of Science, Akdeniz University, Antalya 07070, Türkiye; aslikacar2817@gmail.com (A.K.); zekiyeerdogan0612@gmail.com (Z.E.); 2 Department of Organic Farming, Manavgat Vocational School, Akdeniz University, Antalya 07070, Türkiye; gokhanerdogan@akdeniz.edu.tr (G.E.); akabas@akdeniz.edu.tr (A.K.); 3 Independent Researcher, Antalya 07050, Türkiye; hayriustun07@gmail.com; 4 Department of Agriculture, Nutrition, and Food Systems, University of New Hampshire, Durham, NH 03824, USA; 5 Vocational School of Food, Agriculture and Livestock, Burdur Mehmet Akif Ersoy University, Burdur 15100, Türkiye

**Keywords:** drought stress, grafting, cucumber (*Cucumis sativus*), rootstock–scion compatibility, proline accumulation, stress tolerance

## Abstract

Drought is one of the major abiotic stress factors limiting crop yield and quality, posing a significant threat to sensitive species like cucumber (*Cucumis sativus* L.). This study evaluated the potential of bitter melon (*Momordica charantia* cv. Nusret F_1_) and squash (*Cucurbita pepo* cv. Aygır F_1_) as rootstocks to improve drought-related performance in cucumber scions (Akıncı F_1_ and Baymali F_1_). Grafted and non-grafted plants were grown under two irrigation regimes for 21 days: well-watered control (100% field capacity) and a water-withholding drought treatment. Drought stress significantly reduced morphological parameters across most experimental groups. Under drought conditions, the Akıncı F_1_/Aygır F_1_ combination showed the highest proline accumulation. This biochemical response was accompanied by pronounced reductions in dry leaf and dry biomass. This pattern suggests that proline accumulation is more closely associated with stress severity than with growth maintenance under drought conditions. Conversely, the Baymali F_1_/Aygır F_1_ combination maintained relatively higher leaf dry weight under drought, suggesting better growth maintenance under drought. Plants grafted onto Nusret F1 generally produced the lowest biomass but showed enhanced proline synthesis, indicating a stronger stress response despite reduced growth. In conclusion, while Aygır F_1_ supports higher growth and biomass maintenance under well-watered conditions, drought responses are strongly influenced by scion-rootstock compatibility and distinct physiological strategies, highlighting the importance of distinguishing growth performance from biochemical stress indicators such as proline accumulation.

## 1. Introduction

Plants are continuously subjected to fluctuating environmental conditions and various abiotic stress factors, such as water scarcity, salinity, excessive light, extreme temperatures, and nutrient deficiencies, all of which adversely affect plant productivity [1]. Among these stress factors, drought remains one of the most critical challenges to global crop production. Under limited water availability, plants experience profound morphological and physiological disruptions, including reduced cell expansion, loss of turgor, impaired photosynthetic carbon assimilation, and substantial reductions in leaf mass [2,3]. These stress conditions trigger excessive production of reactive oxygen species (ROS), which induces lipid peroxidation and disrupts cellular homeostasis [3,4]. To cope with such adverse conditions, plants undergo complex physiological and biochemical adjustments, including enhanced abscisic acid (ABA) synthesis and increased accumulation of sugars and proline [5,6].

Cucumber (*Cucumis sativus* L.), an economically important vegetable crop worldwide, is particularly sensitive to water deficit due to its shallow root system and high transpirational demand [7]. In commercial production systems, grafting is widely used in cucurbits to improve water and nutrient acquisition, enhance root system vigor, and increase stress tolerance under suboptimal environmental conditions [8]. Even transient drought stress can significantly reduce vine elongation, leaf area development, and fresh biomass accumulation, thereby negatively affecting yield and fruit quality [9]. From a physiological perspective, drought stress reduces photosynthetic performance and membrane integrity [10], prompting the rapid accumulation of osmotic regulators such as proline, soluble sugars, and phenolic compounds as adaptive mechanisms [11].

Developing effective strategies to ensure sustainable vegetable production under drought stress is therefore critical. Although conventional breeding approaches remain essential, their progress is often inherently slow. Grafting has emerged as a promising alternative, offering a rapid and efficient method for producing plants with enhanced tolerance to changing environmental conditions [12]. Grafting techniques were first introduced in Korea and Japan in the late 1920s for watermelon cultivation and were later extended to other cucurbit and solanaceous crops, including cucumber. By combining stress-tolerant rootstock with a high-yielding, high-quality scion to form a single biological unit, grafting offers a highly viable solution to challenges related to climate change and water scarcity in intensive production systems [13]. The success of grafting largely depends on rootstock traits and scion–rootstock compatibility, and its use has increased substantially in vegetable crops, particularly within the *Cucurbitaceae* and *Solanaceae* families [14].

Bitter melon (*Momordica charantia* L.) is a member of the *Cucurbitaceae* family, known by various names, including karela, balsam pear, and bitter gourd [15]. This species is widely distributed in tropical and subtropical regions worldwide [16]. In Türkiye, its cultivation occurs predominantly in the regions of Bursa, Yalova, and Antalya [17]. Previous studies have shown that bitter melon can serve as a stress-tolerant rootstock, improving scion performance under various abiotic stresses, such as heat [18] and low temperature [19], which has been associated with enhanced photosynthetic efficiency and maintaining cellular redox balance. *Momordica* genotypes can increase water uptake efficiency by creating a deep root system with a large root volume under water-limited conditions [20]. It has been found that grafting cucumber scions onto *Momordica* rootstocks does not result in any structural incompatibility of vascular tissues; consequently, it is hypothesized that it may help maintain photosynthetic performance and biomass production of the scion, potentially through the induction of hormonal signaling such as root-derived cytokinins [21,22]. Therefore, *Momordica* rootstock, with its unique root characteristics and physiological compatibility, presents a potential alternative rootstock for drought-sensitive cucumber genotypes. *Cucurbita pepo* (squash) rootstocks are widely used commercially due to their strong root systems, high graft compatibility, and reported tolerance to soil-borne diseases and various environmental stresses [23]. In contrast, the potential of *Momordica charantia* as a drought-responsive rootstock remains less explored, particularly regarding its graft compatibility with cucumber scions and its possible contribution to plant performance under water-limited conditions.

Therefore, the aim of this study was to investigate the graft compatibility and rootstock suitability of bitter melon and squash rootstocks for cucumbers, evaluating how rootstock–scion combinations influence morphological, physiological, and biochemical responses under drought stress conditions. We hypothesized that (i) squash would enhance biomass maintenance under drought, while (ii) bitter melon would alter biochemical stress response through osmotic regulation.

## 2. Materials and Methods

### 2.1. Plant Material and Grafting

In this study, the bitter melon genotype Nusret F_1_ (Yüksel Seed Company, Antalya, Türkiye) and the squash genotype Aygır F_1_ (Argeto Seed Company, Antalya, Türkiye) were used as rootstocks. Two cucumber genotypes, Akıncı F_1_ and Baymali F_1_ (Argeto Seed Company, Türkiye), were used as scions. In the experimental design, the cucumber cultivars used as scions were also self-grafted, and non-grafted plants were included as controls (Table 1).

Rootstock seeds (*Momordica charantia* cv. Nusret F_1_ and *Cucurbita pepo* cv. Aygır F_1_) and cucumber scion seeds (*Cucumis sativus* cv. Akıncı F_1_ and Baymali F_1_) were sown on 17 and 20 February 2025, respectively. Grafting was performed 34 days after sowing (DAS) for all genotypes when seedlings were at a similar developmental stage (2–3 true leaves and comparable hypocotyl diameter).

One-cotyledon splice grafting: In this widely used technique for grafting cucurbit vegetables, the hypocotyl diameters of the rootstock and scion must be similar. During grafting, one cotyledon leaf of the rootstock is removed, and an oblique cut approximately 1 cm in length is made on the hypocotyl at an angle of 35–45°, positioned away from the remaining cotyledon. The scion is cut in the same manner, approximately 1 cm below its cotyledon leaves. The cut surfaces of the rootstock and scion are aligned, joined, and secured with a grafting clip. The grafted plants were placed in an adaptation chamber maintained at 25 °C, 95–100% relative humidity, and partial shade for seven days [24,25]. The grafted plants were then transferred to the seedling greenhouse at Akdeniz University, Türkiye.

Seedlings were transplanted into pots, and drought stress was applied one week later at the 2–3 true leaf stage after transplanting. Drought stress was maintained for 21 days, and plants were harvested at the end of the treatment period for final measurements. The experiment was designed as a completely randomized design with six biological replicates for each treatment (control and drought) and each genotype, making a total of 12 plants per genotype. Each pot contained a single plant, and each plant was considered an independent biological replicate. To ensure uniform growth conditions and avoid positional effects, the pots were randomly rearranged every week during the experiment. All plants received the same amount of nutrients. Prior to the drought treatment, plants were irrigated twice per week with Hoagland nutrient solution [26]. The electrical conductivity (EC) of the solution was adjusted to 1.5 dS m^−1^, and the pH was maintained at 6.0 to provide optimal nutrient conditions for the commercial peat substrate. Drought severity was assessed using a morphological wilting-based criterion, defined as permanent loss of leaf turgor, visible apical shoot bending, and cessation of new leaf emergence. This visual scoring system was used as a proxy for severe drought stress in the absence of direct physiological measurements.

### 2.2. Experimental Design

Seedlings were transplanted into pots (15 cm in diameter by 14 cm tall) filled with commercial peat (Humin substrate N3, Klasmann-Deilmann, Geeste, Germany), with six individual plants (biological replicates) per treatment (control and drought) and genotype, totaling 12 plants per genotype. Plants were placed in a greenhouse at Akdeniz University, Türkiye. Drought stress was initiated one week after transplantation. Two irrigation regimes, control (C) and drought (D), were applied to the plants. Control plants were irrigated twice per week to field capacity (100%), while the drought stress group received no irrigation. To ensure uniform growth conditions, pots were randomly rearranged weekly throughout the experiment.

The experiment was conducted over a 21-day drought period following the initiation of drought stress. During this period, water was withheld completely, and no supplemental soil moisture monitoring was performed. Drought stress was imposed using a standardized water-withholding treatment to compare genotype responses. Direct soil moisture measurements were not included, and drought severity was defined using a consistent visual wilting criterion applied uniformly across all genotypes. The stress treatment was maintained until clear physiological and morphological differences were observed among treatments.

### 2.3. Morphological Evaluation

The growth parameters (plant height, stem diameter, and number of leaves) were measured weekly throughout the experiment. For the number of leaves, all fully expanded leaves were counted, and the cotyledons were not considered.

Root Extraction and Length Measurement: At the end of the experiment, pots were inverted and emptied. The root system was carefully washed to remove substrate residues, then extended for measurement, and maximum root length was measured using a standard laboratory ruler. Root length measurements were performed following the method of Kabaş et al. [27]. Care was taken during root washing to minimize the loss of fine roots prior to measurement. At harvest, additional growth parameters, including leaf, stem, and root fresh and dry weights, were measured for all plants. Roots, stems, and leaves were collected separately and weighed to determine fresh weight (FW). Samples were oven-dried at 65 °C for 48–72 h until constant weight and then weighed to determine dry weight (DW), which was used to calculate water content.

### 2.4. Biochemical Analyses

To evaluate the osmotic and oxidative stress responses, free proline and MDA were quantified for each sample using the youngest fully expanded leaf. For each experimental group, six biological and two technical replicates were used. Briefly, 200 mg of fresh leaf tissue was homogenized in 1 mL of 3% 5-sulfosalicylic acid using a mortar and pestle. The reaction mixture was prepared in 2 mL Eppendorf tubes. 400 μL of extract was mixed with 400 μL of ninhydrin and 400 μL of acetic acid, and incubated at 100 °C for 1 h. After the reaction was stopped on ice, 800 µL of toluene was added, and the mixture was vortexed. The test tubes were kept at room temperature for 5 min for phase separation. The absorbance was read at 520 nm. Proline concentration was determined using a standard curve prepared with L-proline (Sigma-Aldrich, St. Louis, MO, USA) and expressed as µg g^−1^ fresh weight (FW) [28].

Malondialdehyde (MDA) content was measured according to the thiobarbituric acid (TBA) assay. For each experimental group, six biological and two technical replicates were used. Briefly, 200 mg of fresh leaf tissue was homogenized in 1 mL of 0.1% trichloroacetic acid (TCA) and centrifuged at 10,000× *g* for 20 min at 4 °C. The supernatant (1 mL) was mixed with 2.5 mL of 0.5% TBA in 20% TCA and incubated at 95 °C for 15 min. The mixture was then cooled on ice and centrifuged at 4000× *g* for 10 min. Absorbance of the supernatant was recorded at 450 nm, 532 nm, and 600 nm against a blank containing only the TBA reagent. The MDA content was calculated according to the following equations: C (μmol) = 6.45 (A_532_–A_600_) − 0.56 A_450_; where C represents the MDA concentration [29]. Results were normalized and expressed on a fresh weight (FW) basis as µmol per gram of FW (µmol g^−1^ FW).

### 2.5. Statistical Analyses

All statistical analyses were performed in R (version 4.3). Treatment (control vs. drought) and graft combination were included in the model as fixed factors. Given the experimental design, grafted and non-grafted plants were treated as a single factor (graft combination), representing biologically meaningful scion–rootstock pairings rather than independent scion and rootstock factors. The data were analyzed using a two-way ANOVA including treatment, graft combination and their interaction. When the interaction term was significant (*p* < 0.05), mean separation was conducted using Tukey’s HSD test based on the treatment × graft interaction. Prior to analysis, normality was assessed using the Shapiro–Wilk test and homogeneity of variances was verified using Levene’s test. Standard errors were calculated for each group, and bar plots with error bars and Tukey group letters were generated using the ggplot2 package. Pearson correlation coefficients (|r|) were represented by the size of the circles (larger circle indicating stronger correlation) and by the color scale (shades of blue indicating positive correlation and shades of red indicating negative correlation). Correlations that were not statistically significant (*p* > 0.05) were not displayed. Principal component analysis (PCA) was conducted to explore multivariate patterns among the measured morphological and biochemical traits under control and drought conditions. Individual PCA biplots were generated for the full dataset (colored by treatment) and for the Akıncı F_1_ and Baymali F_1_ combinations separately. The biplots included variable loadings represented as arrows and convex hulls to illustrate the distribution of individual observations within each treatment group. Multi-trait radar plots were generated in R using the fmsb package. For multivariate visualization, mean values of each treatment × genotype combination were calculated prior to normalization. To enable comparison across traits measured on different scales and units, all numeric variables were scaled to a 0–1 range using min–max normalization. All measured morphological and biochemical traits were included in the performance index, as each trait represents a distinct and relevant dimension of plant performance under drought stress. Since there was no objective biological criterion for differential weighting, equal weight was applied to all characteristics. The radar charts were constructed as an exploratory visualization tool to facilitate comparison of overall performance profiles across graft combinations.

*X_norm_* = (*X* − *X_min_*)/(*X_max_* − *X_min_*)

For traits where lower values indicated superior performance (e.g., MDA and proline), reverse scaling was applied:

*X_norm_* = (*X_max_* − *X*)/(*X_max_* − *X_min_*)

Proline and MDA were reverse-scaled in the performance index because, in the context of this study, elevated levels of these compounds were associated with increased stress intensity rather than superior performance. When no variation was present (i.e., $X_{max}=X_{min}$), a constant value of 0.5 was assigned. A performance index was computed as the sum of normalized trait values for each Treatment × Genotype combination. Based on this index, the highest- and lowest-performing combinations were identified and visualized using radar charts.

## 3. Results

### 3.1. Biomass Accumulation (Dry and Fresh Weights)

**Figure 1:**
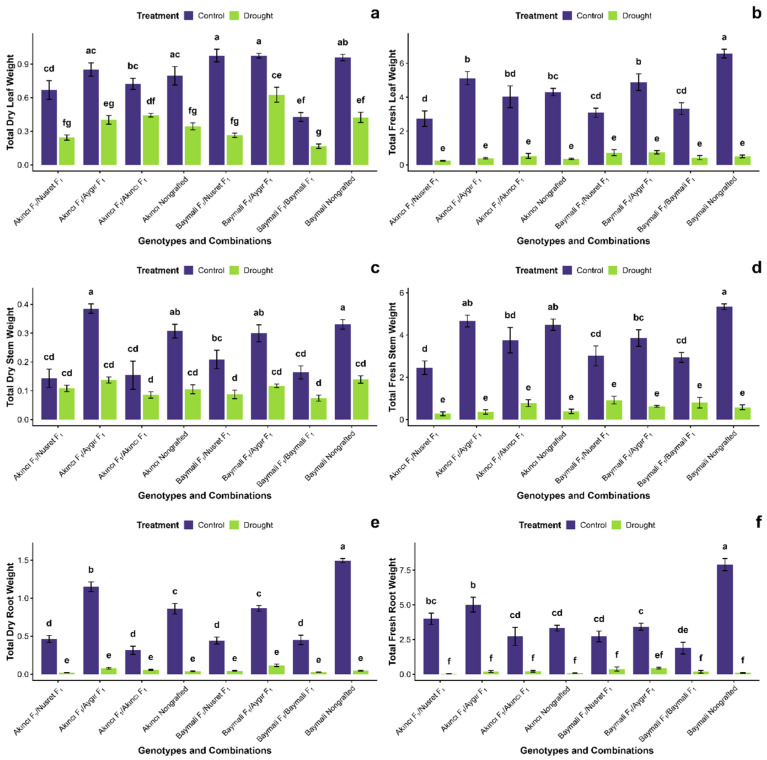
Morphological traits of grafted and non-grafted cucumber plants under control and drought conditions. (**a**) Dry leaf weight, (**b**) Fresh leaf weight, (**c**) Dry stem weight, (**d**) Fresh stem weight, (**e**) Dry root weight, (**f**) Fresh root weight. Letters indicate significant differences among treatments × graft combinations according to Tukey’s HSD test at *p* < 0.05. Data are presented as mean ± standard error (*n* = 6).

Drought significantly reduced fresh and dry biomass in leaves, stems, and roots across most graft combinations, while a significant Treatment × Genotype interaction indicated that the magnitude of this reduction was graft-dependent (Figure 1). Under control conditions, Aygır F_1_-grafted and non-grafted combinations generally accumulated the highest leaf, stem, and root biomass. whereas Nusret F_1_-grafted combinations consistently produced the lowest values. Fresh biomass was significantly reduced by drought in all combinations, and no combination was able to sustain biomass accumulation at levels comparable to the respective control plants.

Plants grafted onto Nusret F_1_ consistently showed lower root biomass compared with those grafted onto Aygır F_1_. Therefore, root biomass declined sharply under drought in all genotypes, with no combination maintaining root weight comparable to its respective control.

### 3.2. Growth Parameters (Plant Height, Leaf Number, and Root Length)

**Figure 2:**
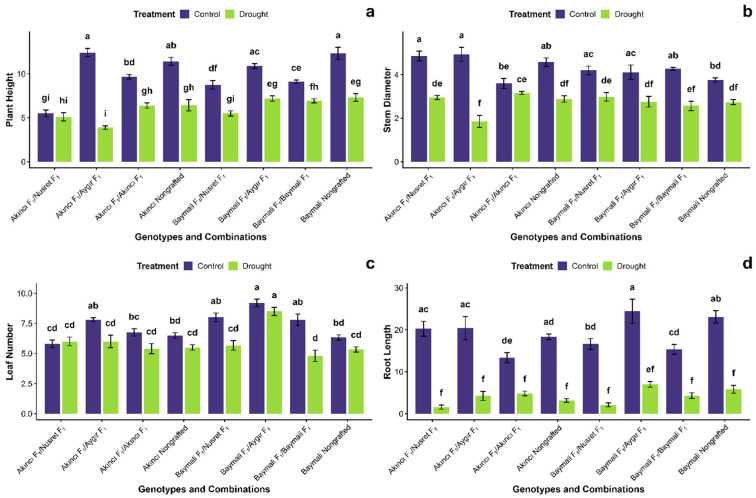
Growth parameters of grafted and non-grafted cucumber plants under control and drought conditions. (**a**) Plant height, (**b**) Stem diameter, (**c**) Leaf number, (**d**) Root length. Letters indicate significant differences among treatments according to Tukey’s HSD test at *p* < 0.05. Data are present as mean ± standard error (*n* = 6).

A decrease in growth parameters was generally observed under drought conditions (Figure 2). Aygır F_1_-grafted and non-grafted plants reached the greatest height under control conditions, similar to non-grafted controls of Akıncı F_1_ and Baymali F_1_. However, this advantage was largely lost under drought. Root length decreased markedly under drought in every combination, with self-grafted Akıncı F_1_ and Baymali F_1_ showing the shortest roots even under control conditions.

### 3.3. Biochemical Stress Indicators

**Figure 3:**
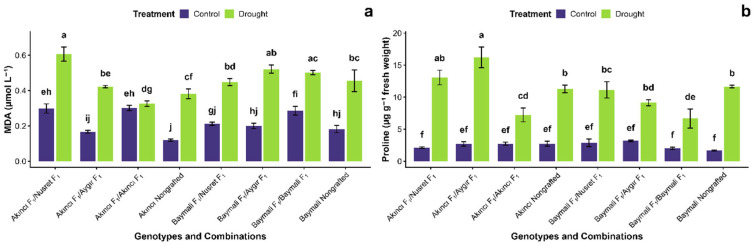
Biochemical stress indicators of grafted and non-grafted cucumber plants under control and drought conditions. (**a**) MDA and (**b**) Proline. Letters indicate significant differences among treatments according to Tukey’s HSD test at *p* < 0.05. Data are present as mean ± standard error (*n* = 6).

Drought significantly increased both MDA and proline content across genotypes, confirming that water deficit induced oxidative stress and a parallel biochemical stress response (Figure 3). The Akıncı F_1_/Nusret F_1_, Baymali F_1_/Aygır F_1_, and Baymali F_1_/Baymali F_1_ graft combinations exhibited the highest MDA concentrations. In contrast, the self-grafted Akıncı F_1_/Akıncı F_1_ plants maintained comparatively lower MDA levels, which were statistically similar to those of the non-stressed control.

Proline concentrations increased significantly in all genotypes subjected to drought stress compared with their respective controls, but the response was strongly graft-dependent. Following the 21-day water withholding, the highest proline levels were observed in Akıncı F_1_ scions regardless of rootstock (Aygır F_1_ or Nusret F_1_). In contrast, the lowest proline accumulation was detected in Baymali F_1_/Aygır F_1_, Baymali F_1_/Baymali F_1_, and Akıncı F_1_/Akıncı F_1_ plants. Notably, self-grafted plants generally accumulated less proline than heterografted combinations. This suggests that the magnitude of this stress response depends on scion–rootstock pairing rather than genotype alone.

### 3.4. Multi-Trait Evaluation of Scion-Rootstock Combinations: Insights from Morphological and Biochemical Performance

**Figure 4:**
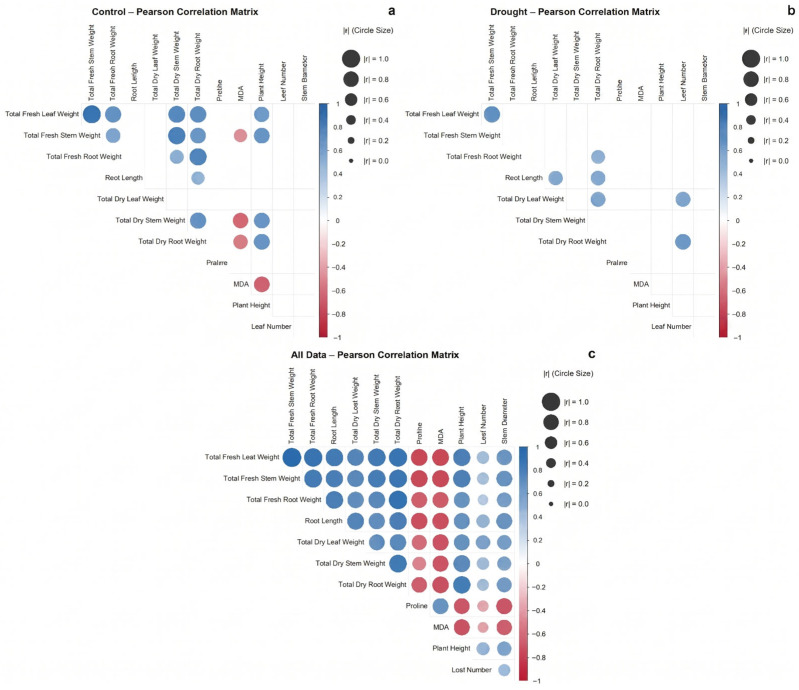
Pearson correlation matrices for different conditions for (**a**) control, (**b**) drought and (**c**) both conditions together. Circle size indicates the strength of correlation, while color represents direction (blue = positive, red = negative). Non-significant correlations (*p* > 0.05) are not displayed.

**Figure 5:**
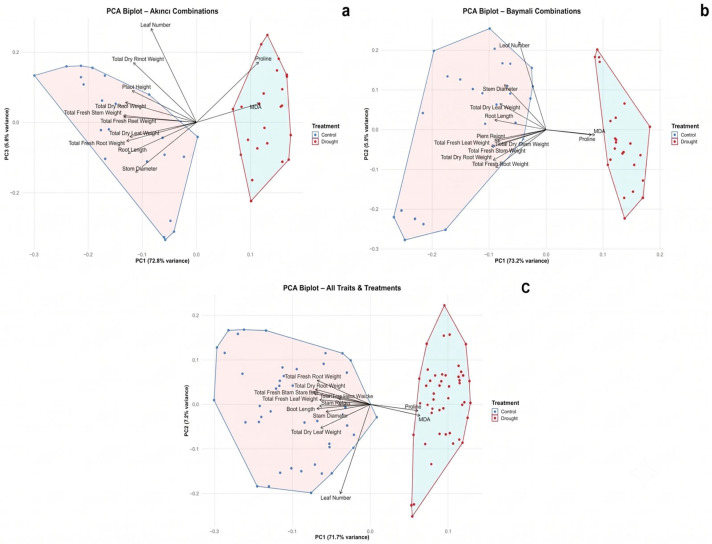
Principal component analysis (PCA) biplots of morphological and biochemical traits for different graft combinations: (**a**) Akıncı F_1_, (**b**) Baymali F_1_, and (**c**) combined genotypes. Points are colored by treatment (control = blue; drought = red). Arrows represent variable loadings, indicating the direction and magnitude of each trait’s contribution to the principal components. The proximity of points reflects similarity among samples, while the angle between vectors indicates correlations among traits.

Correlation analyses revealed that under control conditions, most of the biomass-related traits were strongly and positively associated with one another (Figure 4a). Conversely, a negative correlation was observed between MDA and several vegetative parameters, namely plant height, total dry stem weight, total dry root weight, and total fresh stem weight. Interestingly, this negative correlation was not observed under drought conditions (Figure 4b). When the entire dataset was examined, both proline and MDA showed patterns opposite to the biomass measurements, displaying negative correlations with all growth biomarkers while showing positive correlations with each other (Figure 4c). When all genotypic combinations were evaluated together, PCA revealed that PC1 and PC2 explained 71.7% and 7.3% of the total variance, respectively. In the biplot, proline and MDA vectors were oriented towards the drought stress group. In contrast, growth- and biomass-related traits were positioned closer to the control group (Figure 5c).

When Akıncı F_1_ and Baymali F_1_ combinations were evaluated independently, proline and MDA vectors showed greater separation along PC1 within the Akıncı F1 combinations. Additionally, total leaf number showed a relatively distinct orientation in the biplot and displayed weaker associations with the other biomass-related traits (Figure 5a,b).

The PCA revealed the effects of rootstocks on drought response. Control and drought-treated plants were clearly separated along PC1. Biomass-related traits were associated with control plants, whereas proline and MDA were associated with drought-treated plants, indicating that these biochemical traits were closely linked to drought stress under the experimental conditions. However, some scion–rootstock combinations, such as Baymali F1/Aygır F1, were positioned closer to the control group and maintained higher biomass under drought. In contrast, Akıncı F1/Nusret F1 showed higher proline and MDA levels together with lower biomass and clustered more closely with the drought group. These results suggest that the beneficial effects of certain rootstocks under drought are mainly reflected in improved biomass maintenance rather than reduced accumulation of stress-related biochemical markers.

**Figure 6:**
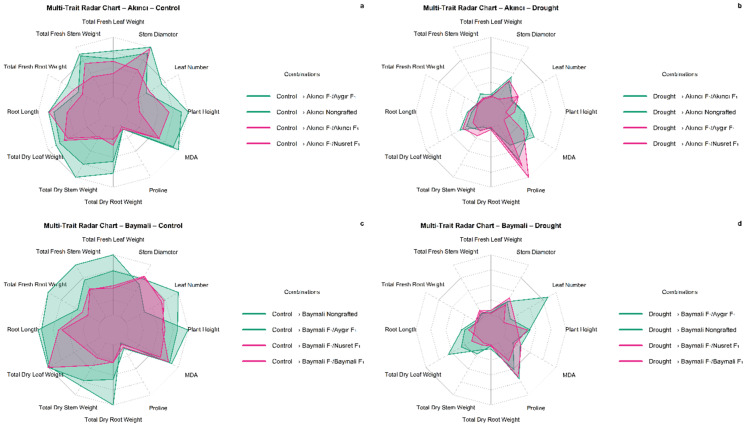
Multi-trait radar charts showing the performance of Treatment × Genotype combinations: (**a**) Akıncı F_1_ under control conditions, (**b**) Akıncı F_1_ under drought conditions, (**c**) Baymali F_1_ under control conditions, and (**d**) Baymali F_1_ under drought conditions. Green shading indicates higher trait values, whereas pink shading indicates lower trait values across the evaluated parameters.

Multi-trait radar plots demonstrated the morphological differences among graft combinations (Figure 6). The Akıncı F_1_/Aygır F_1_ graft displayed superior morphological vigor under control conditions, maximizing root and stem biomass (Figure 6a). Similarly, the Baymali F_1_/Aygır F_1_ combination outperformed other Baymali F_1_ combinations under optimal watering (Figure 6c). Under drought stress, Aygır F_1_ continued to show morphological benefits; meanwhile, the Baymali F_1_/Aygır F_1_ combination performed better than its non-grafted counterpart (Figure 6b,d). Conversely, the Nusret F_1_ rootstock combinations consistently exhibited the lowest overall growth performance under both conditions.

## 4. Discussion

### 4.1. Morphological Responses and Rootstock Efficiency

Drought stress severely restricted vegetative growth in this study, resulting in significant reductions in the fresh and dry biomass of leaves, stems, and roots across all genotypes. This generalized reduction is likely associated with decreased cell expansion and turgor loss, which impair cell elongation and division and, in turn, restrict photosynthetic carbon assimilation [30,31]. Physiological responses to these growth reductions are thought to be associated with alterations in root hydraulic conductivity and root-derived signal transduction pathways. The reduction in stem biomass under drought may be attributed to restricted cell expansion and the development of vascular tissues, both of which affect nutrient transport and the formation of supporting tissues [32,33]. This structural limitation may restrict long-distance nutrient transport and upward water movement in the xylem, thereby directly disrupting water relations in the grafting region. Similarly, the dramatic decrease in root length and mass under stress may reflect the inhibition of root meristematic activity due to mechanical impedance and reduced turgor in the soil-root interface [34,35,36].

The results highlighted a distinct difference in the vigor conferred by the two rootstock species. The squash rootstock (Aygır F_1_) generally supported superior vegetative growth under well-watered conditions, which may reflect its vigorous root system architecture [25,37]. In contrast, plants grafted onto bitter melon (Nusret F_1_) exhibited consistently lower biomass and shorter plant height. While rootstock-induced scion vigor is well-documented phenomenon, the generalized growth reduction observed with Nusret F_1_ may indicate sub-optimal physiological or anatomical compatibility at the graft union [38,39]. Structural bottlenecks at the graft interface may restrict symplastic and apoplastic translocation of dissolved substances, vascular continuity, and bulk water flow. These limitations may reduce the scion developmental capacity, even under optimal moisture conditions.

### 4.2. Scion-Rootstock Interaction and Stress Sensitivity

The efficacy of grafting in mitigating drought stress was strongly dependent on the specific scion-rootstock combination. The Akıncı F_1_/Aygır F_1_ combination demonstrated higher phenotypic plasticity; it produced the highest biomass under optimal conditions but suffered severe reductions under drought. This significant decline suggests that while vigorous rootstocks like Aygır F_1_ can enhance water uptake and promote superior vegetative growth under ideal conditions [40,41], they may not suffice to protect highly demanding scions from severe dehydration during prolonged stress. During periods of severe water stress, aggressive root systems may initiate robust root-stem signaling mechanisms. In these processes, ABA synthesized in the roots may be transported via the xylem, resulting in rapid stomatal closure and a more compact stomatal arrangement [42].

Although endogenous hormone levels were not directly quantified in the present study, previous studies indicate that such severe drought stress induces hormonal imbalances, particularly a decrease in cytokinin and an increase in ABA levels [43]. These literature-derived pathways provide a reasonable hypothesis that may help explain our phenotypic and vegetative data, where shifts in root-shoot signal transduction potentially contribute to the observed stomatal regulation and growth inhibition under progressive water deficit. Conversely, the Baymali F_1_ scion grafted onto Aygır F_1_ maintained greater stability in leaf number and dry weight under stress. This resilience suggests a more favorable and stable rootstock-scion interaction for biomass maintenance. In compatible grafts, enhanced root-to-shoot signaling efficiency may facilitate better biomass allocation and vascular conductivity, helping to sustain shoot development even under stress [42]. Furthermore, the consistently lower growth performance of the Nusret F_1_ combinations under both conditions suggests a sub-optimal physiological compatibility with these specific cucumber scions. Taken together, these findings reinforce that grafting success is not solely determined by the rootstock’s inherent genetic vigor but is fundamentally determined by the synergistic compatibility between the rootstock and the specific scion genotype.

In the present study, these explanations remain theoretical models based strictly on phenotypic and biomass responses. Therefore, a comprehensive physiological validation study should also be conducted. To experimentally test and validate these hypotheses regarding adaptation, particularly under water-deficient conditions, future research involving histological sections of the graft union, direct stomatal and photosynthetic assessments, and targeted hormone measurements (such as ABA and cytokinins) is of vital importance.

### 4.3. Biochemical Mechanisms: Oxidative Stress and Osmotic Adjustment

Under drought conditions, plants frequently experience severe oxidative stress characterized by the excessive generation of reactive oxygen species (ROS), which induces lipid peroxidation in cellular membranes and results in the accumulation of MDA, a primary biomarker of oxidative damage. In our study, the elevated MDA levels in the Akıncı F_1_/Nusret F_1_ and Baymali F_1_/Aygır F_1_ combinations suggest that these specific graft pairings suffered more intense cellular disruption under severe drought, serving as physiological markers of oxidative stress severity rather than structural resilience. While grafting has been reported to alleviate this damage by enhancing antioxidant enzyme activities [39,41], specifically triggering ROS scavenging mechanisms. The differing MDA patterns observed here highlight that rootstock selection alone may not guarantee oxidative stress mitigation if the overall scion–rootstock interaction is physiologically mismatched or anatomically stressed. This highlights that mitigating lipid peroxidation is a systemic, emerging feature of a highly compatible graft combination, rather than an automatic trait independently provided by the parental genotype.

In addition to antioxidant defense, osmotic adjustment represents a major physiological mechanism for drought adaptation. Although this process is conceptually understood to rely on active accumulation of dissolved substances that reduce cellular osmotic potential and maintain an appropriate water potential gradient during water deficiency, the present study did not directly measure these specific biophysical parameters. A reduction in osmotic potential is generally considered critical for turgor conservation and may contribute to maintaining stomatal function under water deficit; however, these parameters were not directly evaluated in the present study. However, these pathways remain experimental frameworks within the context of the present study. This speculative nature is further highlighted when interpreting proline accumulation under water stress, which remains challenging, as proline may function either as an active component of drought tolerance mechanisms or as a passive biomarker of severe stress damage [44,45]. In this study, this ambiguity was addressed by examining contrasting biomass outcomes among different hybrid combinations. The Akıncı F_1_/Aygır F_1_ and Akıncı F_1_/Nusret F_1_ combinations exhibited the highest proline accumulation under drought, but also experienced one of the most pronounced reductions in dry leaf and dry stem weights. This physiological distinction offers preliminary evidence that elevated proline content in specific combinations does not adequately sustain growth. This finding underscores the necessity for direct physiological validation, including tissue water potential monitoring and cell osmometry, to experimentally confirm these adaptive strategies.

Additionally, the strong positive correlation observed between proline and MDA in the dataset supports the interpretation that increased proline levels during progressive drought do not indicate successful adaptation. Instead, they may function as parallel stress indicators, suggesting that physiological stress is advanced and severe cellular dehydration has occurred [39,46]. Therefore, the observed proline accumulation may represent a metabolic consequence of stress-induced cellular disruption rather than directly conferring drought tolerance. This interpretation is further supported by our correlation and principal component analysis. The positive statistical correlation observed between proline and MDA levels under drought conditions indicates that both increase concurrently as stress severity intensifies. In the PCA, PC1 explained 71.7% of the variance and separated control plants from drought-stressed plants. Biomass traits were grouped with the control plants, while proline and MDA were grouped together with the drought-stressed plants (Figure 4c and Figure 5c). Because proline increased together with the cell-damage marker MDA rather than with maintained growth, the multivariate results do not strongly support osmotic regulation as the main driver of rootstock-conferred tolerance. Rather than implying a direct causal biological relationship where proline provides absolute tolerance, this pattern suggests that both molecules serve as parallel indicators of the plant’s physiological distress. The multivariate analysis provides an integrated view of drought responses across morphological and biochemical traits. The separation of drought- and control-treated plants along PC1 indicates that biomass reduction and the accumulation of proline and MDA are coordinated responses to water deficit. The clustering of proline and MDA with drought-treated samples suggests that these biochemical changes are associated with stress severity rather than growth maintenance. In contrast, differences among graft combinations in PCA space indicate that rootstock–scion interactions influence the balance between growth preservation and stress-associated biochemical responses under drought conditions.

Collectively, our morphological and biochemical findings suggest that the effectiveness of grafting may depend on the plant’s ability to translate biochemical stress responses into sustained vegetative growth, potentially reflecting physiological compatibility between the scion and rootstock.

An additional limitation of the present study is that drought severity was defined using a standardized water-withholding protocol and a consistent visual wilting criterion rather than direct measurements of soil moisture or soil water potential. Consequently, the intensity of water deficit is characterized qualitatively rather than quantitatively, which limits direct comparison with studies reporting volumetric soil water content or water potential. Nevertheless, the standardized treatment was applied uniformly across all graft combinations, allowing reliable relative comparisons of genotype responses under the same experimental conditions.

### 4.4. Implications for Postharvest Quality and Trait Transferability

While this study examined the seedling and vegetative stages of cucumber development under short-term drought, the rootstock-induced physiological and biochemical changes may have significant implications for mature fruit quality, storage performance, and post-harvest shelf-life [47]. A critical question in commercial cucumber production is whether genotypes exhibiting better early-stage drought tolerance can maintain high fruit yield and quality at harvest. While robust seedlings and root-mediated hydraulic homeostasis ensure uniform crop establishment under initial water deficits, the subsequent reproductive stages introduce highly complex carbon allocation patterns and resource-pool interactions. During fruit development, the physiological transition from vegetative accumulation to heavy reproductive sink demand drastically alters whole-plant source–sink dynamics. However, the findings, together with previous studies, suggest that superior early root system architecture may contribute to later developmental performance, although hormonal regulation was not directly assessed. Previous studies have reported that improved root-zone efficiency may contribute to reduced fruit abortion and improved fruit quality traits under water deficit [48]. However, these parameters were not evaluated in the present study. Furthermore, since drought stress commonly leads to the accumulation of bitter cucurbitacins in cucumber fruits, genotypes demonstrating effective stress mitigation during the vegetative stage are more likely to reduce these undesirable secondary metabolites in the final product under commercial field conditions [49]. Therefore, our seedling-stage findings represent a critical initial step and provide information for long-term, full-season field trials aimed at thoroughly evaluating yield behavior, fruit quality, and storage performance under water-deficit conditions.

In full-cycle commercial cultivation, pre-harvest growing conditions and stress management protocols are primary determinants of fruit water relations and structural integrity at harvest. In this study, the high vegetative stability and root system viability exhibited by Aygır F_1_ indicate that compatible heterograft combinations might sustain more stable water and mineral uptake during field water deficit. Our results suggest that improved vegetative performance may reflect more effective water relations, which could potentially contribute to fruit turgor maintenance, although hydraulic traits and postharvest characteristics were not directly measured. This stability may contribute to reduced critical post-harvest defects, including water loss through accelerated transpiration, fruit shrinking, loss of turgor, and rubbery texture, all of which reduce the shelf life and marketability of commercial cucumbers [50].

Furthermore, evaluating the transferability of seedling-stage grafting responses to broader agronomic contexts requires a well-defined hierarchy of applicability, governed by strict biological and environmental boundaries. Within the same cultivar group, the transferability of these phenotypic profiles is likely, provided that identical scion–rootstock combinations are employed. When extending to other high-demand cucurbit species, such as melons and watermelons, transferability is moderate and dependent on the confirmed vascular and anatomical compatibility at the graft union. However, recent evidence underscores that agricultural interventions and rootstock-derived benefits do not affect greenhouse vegetable plants uniformly, as primary physiological constraints differ fundamentally among botanical families [8]. For instance, cucumber productivity under abiotic stress is primarily limited by root zone processes, making rootstock selection a highly effective intervention. In contrast, tomato productivity under adverse conditions is mainly constrained by shoot source-receiver relationships [51]. Therefore, although the root-oriented hydraulic advantages of Aygır F_1_ can be largely transferred to plants limited by root zone constraints, its effectiveness may differ when applied to solanaceae greenhouse plants with distinct physiological limitations.

Beyond species-specific constraints, the applicability of these results to different cultivation frameworks is highly dependent. Controlled greenhouse substrate systems optimize rootstock-mediated nutrient and water delivery under uniform management. However, applying these combinations to open-field soil cultivation introduces additional environmental variables, including fluctuating soil mechanical impedance, extreme temperature variations, and altered ambient transpirational demands. Therefore, these findings should not be generalized without careful consideration. Instead, they offer a predictive, conditional framework suggesting that vegetative drought resilience arises from specific scion–rootstock–environment interactions.

The adoption of these rootstocks by commercial growers depends on balancing seed and grafting costs with yield benefits. The Aygır F_1_ demonstrates high compatibility, making it a suitable and economically viable candidate for large-scale commercial cucumber production. In contrast, although the use of bitter melon rootstock is structurally novel, the significant growth losses, reduced biomass, and potentially suboptimal compatibility observed in Nusret F_1_ present considerable financial risks. Based on the growth performance observed in this study, Nusret F1 appears less promising for commercial cucumber production under the tested conditions; however, further evaluation across full production cycles and economic analyses is needed before drawing definitive conclusions. Therefore, selecting rootstocks that improve plant biomass maintenance and stress adaptation may contribute to achieving commercial quality standards in cucumber production under abiotic stress conditions.

## 5. Conclusions

This study demonstrates that grafting cucumber onto bitter melon (*Momordica charantia*) or squash (*Cucurbita pepo*) rootstocks can mitigate drought stress, but effectiveness depends on scion–rootstock compatibility. However, it is important to state clearly that these results apply to young potted plants under short-term controlled drought, and do not necessarily translate directly to full-cycle commercial cucumber production. Aygır F_1_ maximized growth under well-watered conditions, yet the Akıncı F_1_/Aygır F_1_ combination was highly sensitive to drought. Baymali F_1_ grafted onto Aygır F_1_ maintained more stable leaf number and biomass under stress, indicating better drought adaptation. Nusret F1 accumulated high proline under drought but could not maintain growth. Overall, variations in drought tolerance may reflect differences in rootstock–scion compatibility and water-related traits, interacting with scion demand rather than relying on rootstock vigor alone.

## Figures and Tables

**Table 1:** Genotype name, species and experimental design for grafting.

Genotype Name	Species	Combinations	
*Nusret F_1_*	*Momordica charantia*	Akıncı F_1_/Nusret F_1_	Baymali F_1_/Nusret F_1_
*Aygır F_1_*	*Cucurbita pepo*	Akıncı F_1_/Aygır F_1_	Baymali F_1_/Aygır F_1_
*Akıncı F_1_*	*Cucumis sativus*	Akıncı nongrafted	Akıncı F_1_/Akıncı F_1_
*Baymali F_1_*	*Cucumis sativus*	Baymali nongrafted	Baymali F_1_/Baymali F_1_

## Data Availability

The raw data supporting the conclusions of this article will be made available by the authors on request.

## References

[1] Verslues P.E., Agarwal M., Katiyar-Agarwal S., Zhu J., Zhu J.-K. (2006). Methods and concepts in quantifying resistance to drought, salt and freezing: Abiotic stresses that affect plant water status. Plant J..

[2] Akram N.A., Ashraf M., Sadiq M. (2020). Exogenous application of L-methionine mitigates the drought-induced oddities in biochemical and anatomical responses of bitter gourd (*Momordica charantia* L.). Sci. Hortic..

[3] Fathi A., Shiade S.R.G., Saleem A., Shohani F., Fazeli A., Riaz A., Rahimi M. (2025). Reactive oxygen species (ROS) and antioxidant systems in enhancing plant resilience against abiotic stress. Int. J. Agron..

[4] Mittler R. (2002). Oxidative stress, antioxidants and stress tolerance. Trends Plant Sci..

[5] Alagoz S.M., Lajayer B.A., Ghorbanpour M. (2023). Proline and soluble carbohydrates biosynthesis and their roles in plants under abiotic stresses. Plant Stress Mitigators.

[6] Fujita M., Hasanuzzaman M. (2022). Approaches to enhancing antioxidant defense in plants. Antioxidants.

[7] Sari T.A., Chandra B., Rivai H. (2021). Overview of traditional use, phytochemical and pharmacological activities of cucumber (*Cucumis sativus* L.). Int. J. Pharm. Sci. Med..

[8] Fanourakis D., Makraki T., Vlachogiannakis E., Tsaniklidis G., Körner O., Ntatsi G. (2026). Innovations in agronomy and their impact on greenhouse vegetable yields: Species-specific perspectives. Horticulturae.

[9] Smiti K., Mina U., Verma M., Arya L. (2025). Impact of climate extremes and other key abiotic stresses on cucurbits: A systematic review. Vegetos.

[10] Dhanusri V., Devi H.U.N., Sankari A., Djanaguiraman M., Veeranan V., Giridhari A. (2023). Quantifying the effects of drought stress on cucumber genotypes differing in membrane integrity. J. Appl. Hortic..

[11] Zhang Y., He H., Song M., Chen A., Chen M., Lin W., Yang J., Luo D., Ye J., Xu F. (2025). Advances in physiological and molecular mechanisms of cucumber response to low-temperature stress. Horticulturae.

[12] Singh H., Sethi S., Kaushik P., Fulford A. (2020). Grafting vegetables for mitigating environmental stresses under climate change: A review. J. Water Clim. Change.

[13] Coşkun Ö.F. (2023). The effect of grafting on morphological, physiological and molecular changes induced by drought stress in cucumber. Sustainability.

[14] Bie Z., Nawaz M.A., Huang Y., Lee J., Colla G. (2017). Introduction to vegetable grafting. Vegetable Grafting: Principles and Practices.

[15] Beloin N., Gbeassor M., Akpagana K., Hudson J., de Soussa K., Koumaglo K., Arnason J.T. (2005). Ethnomedicinal uses of *Momordica charantia* (Cucurbitaceae) in Togo and relation to its phytochemistry and biological activity. J. Ethnopharmacol..

[16] Basch E., Gabardi S., Ulbricht C. (2003). Bitter melon (*Momordica charantia*): A review of efficacy and safety. Am. J. Health-Syst. Pharm..

[17] Karataş B. (2025). Elastase and Lipoxygenase Inhibitory Effects and Phytochemical Analysis of the Cultured Sample of *Momordica charantia* L.. Master’s Thesis.

[18] Xu Y., Yuan Y., Du N., Wang Y., Shu S., Sun J., Guo S. (2018). Proteomic analysis of heat stress resistance of cucumber leaves when grafted onto *Momordica* rootstock. Hortic. Res..

[19] Mohammadnia S., Haghighi M. (2021). *Momordica charantia*: Introducing a new rootstock for grafted cucumber under low-temperature stress. Adv. Hort. Sci..

[20] Ali A., Ferdosi F.H., Sarwar M., Anjum S., Mushtaq Z., Liaquat M., Abbas M.T., Anees M., Tariq M.R., Ashraf M.I. (2025). Inducing salt stress tolerance in bitter gourd (*Momordica chanrantia*) through seed treatment with chitosan. Front. Plant Sci..

[21] Han S., Shu S., Wang Y., Jahan M.S., Sun J., Guo S. (2022). Cytokinin plays a critical role in bitter gourd rootstock-induced thermotolerance of cucumber. Veg. Res..

[22] Tao M.Q., Jahan M.S., Hou K., Shu S., Wang Y., Sun J., Guo S.R. (2020). Bitter melon (*Momordica charantia* L.) rootstock improves the heat tolerance of cucumber by regulating photosynthetic and antioxidant defense pathways. Plants.

[23] Nazarpour A., Azizi M., Samadi S., Aroiee H., Farhadi A., Muhammad M., Morshedloo M.R. (2025). Rootstock and grafting type affect the growth and oil quality of medicinal pumpkin (*Cucurbita pepo* Var. *styriaca*). BMC Plant Biol..

[24] Lee J.M. (1994). Cultivation of grafted vegetables I. Current status, grafting methods, and benefits. HortScience.

[25] Lee J.M., Oda M. (2002). Grafting of herbaceous vegetable and ornamental crops. Hort. Rev..

[26] Hoagland D.R., Arnon D.I. (1950). The water-culture method for growing plants without soil. Circ. Calif. Agric. Exp. Stn..

[27] Kabaş A., Antar O., Üstün H., Vilanova S., Vicente O., Prohens J. (2026). Morphological and biochemical responses to water stress in *Solanum pimpinellifolium* and *S. lycopersicum* var. *cerasiforme* accessions. Physiol. Mol. Biol. Plants.

[28] Bates L.S., Waldren R.P.A., Teare I.D. (1973). Rapid determination of free proline for water-stress studies. Plant Soil.

[29] Chen W.P., Li P.H., Chen T.H.H. (2000). Glycinebetaine increases chilling tolerance and reduces chilling-induced lipid peroxidation in *Zea mays* L.. Plant Cell Environ..

[30] Anjum S.A., Xie X., Wang L.C., Saleem M.F., Man C., Lei W. (2011). Morphological, physiological and biochemical responses of plants to drought stress. Afr. J. Agric. Res..

[31] Chaves M.M., Flexas J., Pinheiro C. (2009). Photosynthesis under drought and salt stress: Regulation mechanisms from whole plant to cell. Ann. Bot..

[32] Hsiao T.C., Xu L.K. (2000). Sensitivity of growth of roots versus leaves to water stress: Biophysical analysis and relation to water transport. J. Exp. Bot..

[33] Taiz L., Zeiger E., Møller I.M., Murphy A. (2017). Fisiologia e desenvolvimento vegetal. Plant Physiology and Development.

[34] Comas L.H., Becker S.R., Cruz V.M.V., Byrne P.F., Dierig D.A. (2013). Root traits contributing to plant productivity under drought. Front. Plant Sci..

[35] Smith S., De Smet I. (2012). Root system architecture: Insights from *Arabidopsis* and cereal crops. Phil. Trans. R. Soc. B.

[36] Hemati A., Moghiseh E., Amirifar A., Mofidi-Chelan M., Asgari Lajayer B., Vaishnav A., Arya S., Choudhary D.K. (2022). Physiological effects of drought stress in plants. Plant Stress Mitigators.

[37] Ashraf M.F.M.R., Foolad M.R. (2007). Roles of glycine betaine and proline in improving plant abiotic stress resistance. Environ. Exp. Bot..

[38] Rivero R.M., Ruiz J.M., Romero L. (2003). Role of grafting in horticultural plants under stress conditions. J. Food Agric. Environ..

[39] Shehata S.A., Omar H.S., Elfaidy A.G., El-Sayed S.S., Abuarab M.E., Abdeldaym E.A. (2022). Grafting enhances drought tolerance by regulating stress-responsive gene expression and antioxidant enzyme activities in cucumbers. BMC Plant Biol..

[40] Yang L., Xia L., Zeng Y., Han Q., Zhang S. (2022). Grafting enhances plants drought resistance: Current understanding, mechanisms, and future perspectives. Front. Plant Sci..

[41] Kumar P., Rouphael Y., Cardarelli M., Colla G. (2017). Vegetable grafting as a tool to improve drought resistance and water use efficiency. Front. Plant Sci..

[42] Yordanov I., Velikova V., Tsonev T. (2000). Plant responses to drought, acclimation, and stress tolerance. Photosynthetica.

[43] Aloni B., Cohen R., Karni L., Aktas H.A.K.A., Edelstein M. (2010). Hormonal signaling in rootstock–scion interactions. Sci. Hortic..

[44] Szabados L., Savouré A. (2010). Proline: A multifunctional amino acid. Trends Plant Sci..

[45] Hayat S., Hayat Q., Alyemeni M.N., Wani A.S., Pichtel J., Ahmad A. (2012). Role of proline under changing environments: A review. Plant Signal. Behav..

[46] Arteaga S., Yabor L., Díez M.J., Prohens J., Boscaiu M., Vicente O. (2020). The use of proline in screening for tolerance to drought and salinity in common bean (*Phaseolus vulgaris* L.) genotypes. Agronomy.

[47] Khopade R.Y., Sawargaonkar G.L., Rakesh S., Davala M.S., Kishore K.K., Siddam Y., Singh R., Jat M.L. (2025). Vegetable grafting: A scientific innovation to enhance productivity and profitability of tomato growers under climate change. Front. Agron..

[48] Rouphael Y., Schwarz D., Krumbein A., Colla G. (2010). Impact of grafting on product quality of fruit vegetables. Sci. Hortic..

[49] Shang Y., Ma Y., Zhou Y., Zhang H., Duan L., Chen H., Huang S. (2014). Biosynthesis, regulation, and domestication of bitterness in cucumber. Science.

[50] Kyriacou M.C., Rouphael Y., Colla G., Zrenner R., Schwarz D. (2017). Vegetable grafting: The implications of a growing agronomic imperative for vegetable fruit quality and nutritive value. Front. Plant Sci..

[51] Fanourakis D., Makraki T., Spyrou G.P., Karavidas I., Tsaniklidis G., Ntatsi G. (2026). Environmental Drivers of Fruit Quality and Shelf Life in Greenhouse Vegetables: Species-Specific Insights. Agronomy.

